# Formation of the Immunosuppressive Microenvironment of Classic Hodgkin Lymphoma and Therapeutic Approaches to Counter It

**DOI:** 10.3390/ijms20102416

**Published:** 2019-05-15

**Authors:** Donatella Aldinucci, Cinzia Borghese, Naike Casagrande

**Affiliations:** Molecular Oncology, Centro di Riferimento Oncologico di Aviano (CRO) IRCCS, 33081 Aviano (PN), Italy; cpborghese@cro.it (C.B.); naike.casagrande@libero.it (N.C.)

**Keywords:** Hodgkin lymphoma, tumor microenvironment, immune escape, tumor-associated macrophages

## Abstract

Classic Hodgkin lymphoma (cHL) is characterized by a few tumor cells surrounded by a protective, immunosuppressive tumor microenvironment composed of normal cells that are an active part of the disease. Hodgkin and Reed–Sternberg (HRS) cells evade the immune system through a variety of different mechanisms. They evade antitumor effector T cells and natural killer cells and promote T cell exhaustion. Using cytokines and extracellular vesicles, they recruit normal cells, induce their proliferation and “educate” (i.e. reprogram) them to become immunosuppressive and protumorigenic. Therefore, alternative treatment strategies are being developed to target not only tumor cells but also the tumor microenvironment. Here we summarize current knowledge on the ability of HRS cells to build their microenvironment and to educate normal cells to become immunosuppressive. We also describe therapeutic strategies to counteract formation of the tumor microenvironment and related processes leading to T cell exhaustion and repolarization of immunosuppressive tumor-associated macrophages.

## 1. Introduction

Classic Hodgkin lymphoma (cHL) is responsible for 15% to 25% of all lymphomas, and is the most common lymphoma subtype in children and young adults in the Western world [1]. The current cure rate with traditional, combined modality approaches is high, between 70% and 80%. However, cHL relapses or is refractory to therapy in 20%–30% of patients [1]. The standard of care for the treatment of patients with relapsed or refractory disease is salvage chemotherapy followed by autologous stem cell transplantation. However, toxicity from long-term treatment remains a significant problem, and thus new therapeutic agents with novel mechanisms of action and new drug combinations are being developed [2].

Histologically, a low number of malignant cells, collectively termed Hodgkin and Reed–Sternberg (HRS) cells, characterize cHL. HRS cells include small mononucleated Hodgkin cells and large binucleated or multinucleated Reed-Sternberg cells [3,4], which are surrounded by a quantitatively predominant protective microenvironment [5,6]. Although originating from B-lymphoid cells, HRS cells have almost completely lost their classic B cell lineage markers, but express antigens normally associated with T, myeloid, or dendritic cells [7,8]. HRS cells are characterized by the constitutive activation of nuclear factor kappa B (NF-κB), the deregulated expression of activator protein-1, E2A [8], and interferon regulating factor (IRF) 5 [9]. They express CD30 and CD40 (members of the tumor necrosis factor—nerve growth factor receptor family), IRF4 [10] and CD15 (75%–85%) [2,8]. 

Four histological subtypes of cHL have been identified according to the morphological features of HRS cells (multinucleated giant cells, lacunar cells, and pseudosarcomatous cells) and the cellular composition of the TME: nodular sclerosis (~80% of cases), mixed cellularity (15% of cases), and the less common lymphocyte-rich and lymphocyte-depleted subtypes [11]. Mixed cellularity cHL is composed of T- and B-reactive lymphocytes, plasma cells, eosinophils, granulocytes, histiocytes/macrophages, and mast cells, while nodular sclerosis is characterized by a great number of fibroblast-like cells [12]. In socioeconomically developed countries Epstein–Barr virus (EBV) is associated with approximately one third of cases, while in pediatric cHL in Central and South America with low socioeconomic status, the association can be up to 90% [13,14]. EBV contributes to the chronic inflammatory tumor microenvironment (TME) that surrounds and supports HRS cells [14]. HIV-associated cHL is strongly related to EBV infection [14,15], whereas only a part of HIV-unrelated cases are EBV+ [14]. 

Tumors, including cHL, are composed of cancer cells and a variety of normal cells (fibroblasts, endothelial cells, and immune cells) that together with extracellular matrix components form the TME [16]. Tumor cells of cHL have little proliferative capacity, but are clever in manipulating normal cells to their advantage [17]. In particular, HRS cells can recruit normal cells and then “educate” them to become tumor promoters, i.e., cells that have immunosuppressive and pro-angiogenic functions and that protect the tumor from the effects of anticancer therapy. Moreover, they can expand immunosuppressive regulatory T cells (Tregs), inhibit CD8+ cytotoxic T cells, repolarize tumor-associated macrophages (TAMs), and transform fibroblasts into protective cancer-associated fibroblasts (CAFs) [18,19]. For this purpose, HRS cells exploit surface-expressed molecules, secrete soluble factors such as chemokines, and release extracellular vesicles and this is now considered an additional mechanism of intercellular communication [20]. Therefore, a new therapeutic challenge is not only to kill cancer cells but also to counteract TME formation and the immunosuppressive reprogramming of normal cells [21].

This review describes how HRS cells educate normal cells and summarizes therapeutic strategies to counteract TME interactions being tested in preclinical studies or already adopted in the clinic.

## 2. Importance of the cHL Tumor Microenvironment 

Even if HRS cells represent only a small part of the tumor mass, through the building of a well-organized TME they create a highly aggressive malignancy that, without therapy, is rapidly fatal [2]. Several lines of evidence suggest that HRS cells need the TME to survive. The prognostic significance of positron emission tomography, used to determine the stage of cHL, seems to be related to the reduction of the TME rather than of HRS cells [22]. Moreover, when HRS cells metastasize into non-lymphoid organs, they establish in loco a TME in which they can survive and grow [2,23]. Thus, it is not surprising that research is currently focused on the role of the TME in cHL progression, in the hope of discovering new targets for antitumor therapy.

### 2.1. TME Cellular Composition

TME building in cHL is most likely started and carried out by HRS cells but then is supported by the predominant tumor-educated inflammatory and stromal cells. The cHL TME is composed of numerous CD4-positive T cells and a variable number of eosinophils [24], histiocytes/macrophages [24,25,26], a complex network of B cells [27], mast cells [28], plasma cells [29], fibroblasts [30], mesenchymal stromal cells (MSCs) [31], and endothelial cells [32], as well as a rich extracellular matrix [30]. The TME of EBV-associated cHL is composed of immune cells, including cytotoxic T lymphocytes against EBV-infected HRS cells, and is enriched in histiocytes, dendritic cells, and endothelial cells [14]. It is characterized by a higher number of macrophages than in EBV-unrelated cHL [14]. 

HRS cells are often in close contact with small CD4^+^ T cells (the so-called rosetting T cells) expressing CD40L [33,34,35]. CD4^+^ T cells interact with tumor cells through CD40L, CD80, and CD54 [36] and protect them from cytotoxic T cells and natural killer (NK) cells [2]. In HIV-associated cHL, T cells are replaced by spindle-shaped CD163^+^ rosetting macrophages [15].

### 2.2. TME Formation 

HRS cells produce cytokines/chemokines that are directly involved in TME formation [17]. Eosinophils can be recruited by IL-5 [23], CCL5 [37], CCL28 [38], and GM-CSF [23]; mast cells and MSCs by CCL5 [39,40]; T cells (including Tregs) by CCL5, CCL17, CCL22, and CCL20 [37,41,42,43,44]; monocytes by CCL5 [40] or M-CSF (Table 1).

HRS cells produce cytokines that can increase the growth rate of normal cells in the TME (Table 1). IL-7 can increase the growth of Tregs [52]; IL-13, TNF-α, TGF-β, and FGF of fibroblasts [30]; TGF-β and TNF-α of MSCs [40]; and M-CSF of monocytes [40]. HRS cells, by secreting LT-α, activate endothelial cells, and by up-regulating the adhesion molecules ICAM-1, VCAM-1, and E-selectin, they facilitate T cell recruitment and adhesion in cHL lymph nodes [66]. By secreting VEGF, FGF, and TGF-β, they increase human umbilical vein endothelial cell tubulogenesis [32,59]. 

Normal cells educated by HRS cells can assist in building the TME and promote HRS cell growth [17]. In different studies, cHL-conditioned medium induced the secretion of eotaxin [74] and CCL5 [37,79] by fibroblasts, CCL5 by MSCs [40], CCL3 and CCL17 by monocytes [40], and IL-3 by T cells [46]. Eotaxin can recruit both eosinophils and T cells [74]. IL-3, together with IL-5 and GM-CSF, by increasing the surface expression of both CD40L and CD30L in eosinophils [47], can increase HRS cell growth and CCL5 and IRF4 expression [34,85] (Table 1).

HRS cell growth can be promoted by CCR5 ligands (CCL3, CCL4, and CCL5) secreted by T cells, monocytes and tumor-educated MSCs [37,40]; by IL-3 secreted by T cells [48]; by IL-7 secreted by fibroblasts and MSCs [52]; by IL-15 secreted by monocytic/dendritic and endothelial cells [54]; by APRIL secreted by neutrophils [56]; by the membrane-bound and soluble forms of Jagged1 expressed by endothelial cells, smooth muscle cells and macrophages [63,86]; by CD137L expressed by macrophages [87]; by collagen secreted by stromal cells [88] (Table 1).

Other mechanisms involved in TME formation, tumor growth, and drug resistance mediated by the TME have been reviewed elsewhere [5,17,89]. 

### 2.3. TME Composition as a Prognostic Factor

Given the importance of the TME in cHL growth and survival, it has become a focal point for research aimed at discovering new therapeutic targets and prognostic markers. Many researchers have analyzed its cellular composition (CD4, CD8, Tregs, NK cells, TAMs, etc.) and secretion of molecules involved in tumor growth (e.g., cytokines, chemokines, cytokine receptors) and immunosuppression (e.g., PD-1, PD-L1, IDO, MHC-I, MHC-II). Unfortunately, associations between prognosis and cellular levels, especially of T cells and macrophages, have been studied using different approaches or technologies and often generate different results and conclusions. The abundance of eosinophils and mast cells has been associated with poor prognosis, but the results have not been confirmed [24,29,90]. 

T cells are the main cell type in the cHL tumor microenvironment. Most T cells in the TME are CD4^+^ T helper cells and Tregs, while the levels of CD8^+^ cytotoxic T cells and NK cells are usually low [6]. Cader et al. [91] compared the cellular compositions of cHL biopsies and control reactive lymph node and tonsil samples, using a customized time-of-flight mass cytometry panel, and found that the cHL TME is characterized by Tregs and exhausted T-effector (Teff) cells. Newly diagnosed primary cHLs had a simultaneous increase in active PD-1^−^ Th1 Tregs and exhausted PD-1^+^ Th1 Teffs [91]. PD-1 expression in the TME was similar between patients with favorable and adverse outcomes, suggesting that PD-L1 levels, rather than PD-1, are associated with the responses to anti-PD-1 therapy [92] with nivolumab [1,93] and pembrolizumab [94,95].

Roemer et al. [96] found that genetically driven PD-L1 expression and MHC class II positivity on HRS cells predicted a favorable outcome after PD-1 blockade with nivolumab, whereas clinical responses did not depend on MHC class I expression. 

Multiple studies demonstrated that a high number of infiltrating TAMs [69,97], predominantly derived from circulating monocytes [98], and a high absolute monocyte count in peripheral blood correlate with poor cHL prognosis [26,99]. A high number of TAMs predicted shorter survival after chemotherapy [25] likely because PD-L1^+^ TAMs may neutralize the anticancer activity of PD-1^+^ T cells [100] and NK cells [101]. However, other studies demonstrated no association with elevated TAMs, suggesting that it is not a matter of number but rather of types of TAMs. 

## 3. HRS Cell-Mediated Immune Escape

**Figure 1:**
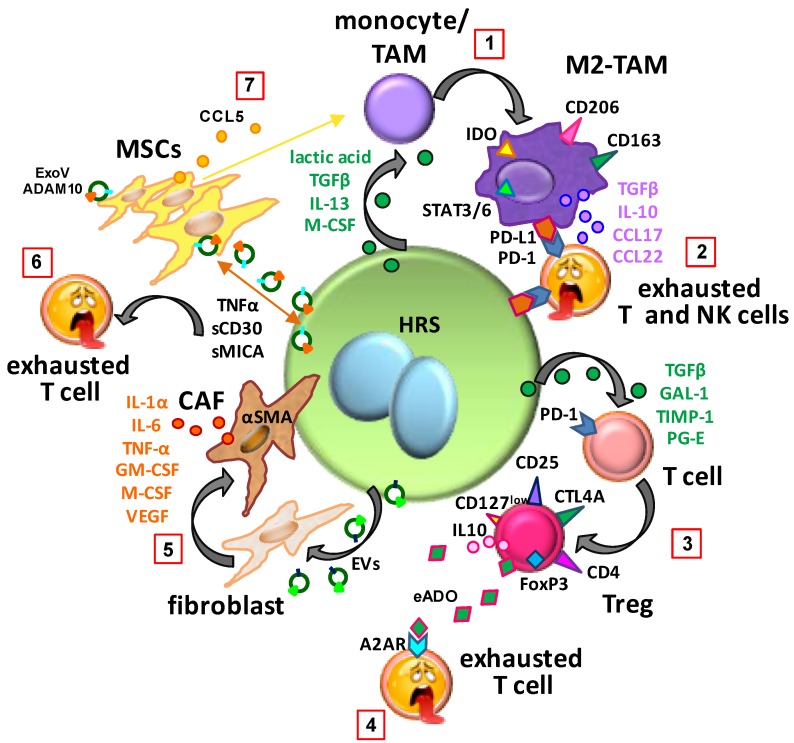
Education of normal cells in the tumor microenvironment of classic Hodgkin lymphoma. (**1**) Hodgkin and Reed–Sternberg (HRS) cells, by secreting transforming growth factor β (TGF-β), interleukin (IL)-13, macrophage colony-stimulating factor (M-CSF) and lactic acid, educate monocytes or tumor-associated macrophages (TAMs) to become immunosuppressive M2-TAMs (programmed death-ligand 1, PD-L1^+^; and indoleamine 2,3-dioxygenase, IDO^+^). (**2**) M2-TAMs, by secreting TGF-β, IL-10, C-C motif chemokine ligand (CCL) 17 and CCL22 and by expressing PD-L1 and IDO, induce exhaustion of programmed cell death protein 1 (PD-1)^+^ effector T and NK cells. (**3**) HRS cells, by secreting TGF-β, galectin-1 (GAL-1), tissue inhibitor of metalloproteinase-1 (TIMP-1) and prostaglandin (PG-E), induce the differentiation of CD4^+^ T cells towards regulatory T cells (Tregs) (forkhead box P3, FoxP3^+^). (**4**) High levels of extracellular adenosine (eADO) in the TME inhibit T effector cell activity. (**5**) Extracellular vesicles (EVs) secreted by HRS cells convert fibroblasts to α smooth muscle actin (αSMA)^+^ cancer-associated fibroblasts (CAFs) that secrete IL-1α, IL-6, tumor necrosis factor (TNF-α), M-CSF, granulocyte-macrophage colony-stimulating factor (GM-CSF), and vascular endothelial growth factor (VEGF). (**6**) The mature, bioactive form of A Disintegrin And Metalloproteinase (ADAM10) is released in exosome-like vesicles (ExoV) by HRS cells and lymph node mesenchymal stromal cells (MSCs). Diffusion of ADAM10 activity due to ExoV results in the release of TNF-α, MHC class I chain-related a (sMICA), and soluble sCD30 that may interfere with host immune surveillance, immunotherapy or brentuximab vedotin activity. (7) HRS cells induce MSC growth and educate MSCs to secrete CCL5. Tumor-educated MSCs (E-MSCs), through the secretion of CCL5, recruit monocytes. CTLA4, cytotoxic T lymphocyte antigen 4; STAT3/6, signal transducer and activator of transcription.

HRS cells can neutralize anticancer immunity by different strategies [102,103]. HRS cells secrete TGF-β [73], IL-13 [53,87], galectin-1 [104], tissue inhibitor of metalloproteinase 1 [105], prostaglandin E2 [106], and lactate [87,107]. These molecules can inhibit Teff functions, expand Tregs, induce the immunosuppressive polarization of TAMs [103], and maintain M2-TAM polarization [107] (Figure 1). 

HRS cells escape from Fas ligand-mediated apoptosis through the overexpression of cFLIP [108]. They also express Fas ligand, and therefore induce apoptosis of activated cytotoxic CD8^+^ T cells [109]. HRS cells escape from immune system recognition by reducing the expression of HLA class I/II [109] and of the NKG2D ligand MHC class I related chain-A(MIC-A) [110]. By inducing the expression of HLA-G [103,111] and HLA-E [109], they protect themselves from the cytotoxic effects of NK cells and T cells. 

CD137 expression, causing the removal of CD137 ligand (CD137L) from tumor cells and antigen presenting cells, inhibits T cell costimulation [87,112]. Another mechanism for escaping antitumor responses is the exhaustion of T cell and NK cell activity through stimulation of PD-1 [113] by PD-L1 expressed on HRS cells [114] and TAMs [101]. 

Recently, the expression of the inhibitory CD200R and BTLA receptors on cHL-infiltrating T cells and of their ligands on HRS cells and immune cells was found to be another mechanism of immune escape [107]. HRS cells, by expressing CD200 and herpes virus entry mediator (HVEM), can suppress T cell activation through CD200-CD200R and HVEM-BTLA interactions [107]. 

## 4. TME-Mediated Immune Escape

Increasing evidence suggests that Tregs [107], as well as MSCs [31] and TAMs contribute to an immunosuppressive TME (Figure 1). MSCs, by modulating NKG2D expression in T cells and its ligand in tumor cells, reduce the immune response to tumor cells [110]. TAMs may exert immunosuppressive activity by expressing PD-L1 [100,101] and indoleamine 2,3-dioxygenase 1 (IDO1), an enzyme that catabolizes tryptophan (Trp) into kynurenine (Kyn) (Figure 1). 

As demonstrated by Carey et al. [100] using a novel multicolor approach to describe the spatial relationship of the cellular components of the cHL TME, PD-L1^+^ TAMs are located closer to PD-L1^+^ tumor cells while PD-1^+^ T cells preferentially localize near PD-L1+ TAMs. From these findings, they proposed a model in which the TME is organized in an “immunoprotective niche”, with PD-L1^+^ TAMs immediately surrounding HRS cells to engage PD-1^+^ T cells or NK cells [101] (Figure 1).

The depletion of tryptophan induces T cell arrest and anergy [115]. Therefore, it is increasingly being recognized as an important micro-environmental factor that suppresses antitumor immune responses, and creates a favorable environment for tumor cells to escape from host immunity [116]. IDO, by converting the essential amino acid tryptophan into various active metabolites such as kynurenin, can inhibit the activity of Teffs and induce those of Tregs. Choe et al. [117] reported that in cHL tissues, IDO was expressed especially by macrophages but not by tumor cells and high levels were associated with inferior survival in cHL patients. 

Another mechanism involved in maintaining an immunosuppressive TME is the production of the anti-inflammatory mediator adenosine (ADO). By binding to its receptor A2AR on Teffs, ADO inhibits their activity [107]. Adenosine is produced by the nucleotide-scavenging ectonucleotidases CD39 and CD73, expressed by Tregs [118]. It is removed by adenosine deaminase, an enzyme that requires CD26 to bind the cell surface. High levels of extracellular adenosine (eADO) in the cHL TME may be due to the downregulation of adenosine deaminase in both HRS and Tregs, thus maintaining an immunosuppressive TME [119] (Figure 1).

## 5. Immunosuppressive Education of Normal Cells in the TME

Normal cells are recruited by HRS cells and then educated to become the immunosuppressive M2-TAM, Tregs or cancer-associated fibroblasts (CAFs). 

### 5.1. Monocyte Polarization Towards M2-TAM

TAMs are distinguished by two types: classically activated macrophages (M1), which promote inflammation, and alternatively activated macrophages (M2), which inhibit inflammation, are immunosuppressive, increase angiogenesis, and activate tumor cells [120]. These two types differ in terms of receptor expression, effector function, and cytokine and chemokine production. M2 macrophage activation of monocytes is induced by M-CSF, IL-4, IL-13, IL-10, TGF-β, glucocorticoid hormones, and vitamin D3, and leads to the secretion of high amounts of IL-10, TGF-β, CCL17, and CCL22 as well as the expression (increased or induced) of CD163, CD206, PD-L1, IDO and pSTAT3/6 [120]. LPS, IFN-ϒ and GM-CSF polarize macrophages towards the M1 phenotype, which induces secretion of IL-1-β, TNF-α, IL-12, IL-18, and IL-23, and increases the expression of CD68, CD80, and CD86 [120]. 

The fact that HRS cells secrete molecules involved in monocyte differentiation into macrophages (M-CSF and/or GM-CSF) and in immunosuppressive polarization (M2-TAM) (TGF-β and IL-13) supports the hypothesis that tumor cells can differentiate TAMs towards an immunosuppressive phenotype (M2-TAM) [17,73]. 

To test if treatment with conditioned medium from cHL cell lines enhanced or maintained the immunosuppressive M2^M-CSF^ phenotype, Tudor et al. [69] used models of the two extreme polarization states of macrophages, namely pro-inflammatory M1 and immunoregulatory M2 macrophages, which were obtained by the stimulation of monocytes with M-CSF and GM-CSF and were referred to as M2^M-CSF^ and M1^GM-CSF^, respectively. They found that HL conditioned medium upregulated both CD163 and CD206 expression in unstimulated peripheral blood monocytes. HRS cells were not able to repolarize M1^GM-CSF^ into M2^M-CSF^ macrophages. A significant inhibition in growth of the cHL cell line L-1236 was found after incubation with conditioned medium from M1^GM-CSF^, but not from M2^M-CSF^ macrophages [69].

Ruella et al. [60] obtained M2 macrophages by culturing monocytes, pretreated with GM-CSF, together with HDLM-2 cells or with HDLM2 conditioned medium. HDLM-2-educated macrophages showed an M2-like phenotype and expressed CD163, CD206, PD-L1, and phosphorylated STAT6. These M2-polarized macrophages inhibited the growth of human CD19 chimeric antigen receptor (CAR) T cells stimulated with CD19^+^ acute leukemia B cells (NALM-6 cells). This evidence suggests that the massive presence of immunosuppressive M2 macrophages in the cHL TME may explain the unsatisfactory results of CAR T cell therapy against the CD30 antigen on HRS cells [121]. Thus, since HRS cells and TAMs express CD123 (IL-3R), in order to target both tumor cells and TAMs a CD123-CART was developed [60].

Recently, a study demonstrated the ability of HRS cells to educate monocytes to become immunosuppressive M2-TAMs [40]. The authors found that treatment of human monocytes with conditioned medium from L-1236 and L-428 cHL cell lines increased the expression of CD206, PD-L1, and IDO. The tumor-educated monocytes (E-monocytes) secreted high amounts of the immunosuppressive cytokines IL-10, TGF-β, and CCL17. Moreover, E-monocyte conditioned medium inhibited the growth of PHA-activated lymphocytes and increased the clonogenic growth of HRS cells [40]. 

Lactic acid secreted by tumor cells was found to favor the M2-like polarization of macrophages [122] (Figure 1). In this context, Locatelli et al. [123] demonstrated that the inhibition of lactic acid production by HRS cells repolarized tumor-promoting M2-like TAMs toward tumor suppressive M1-like TAMs. This has been demonstrated using the PI3Kδ/ϒ inhibitor RP6530 which downregulates the metabolic regulator pyruvate kinase muscle isozyme 2 (PKM2) that catalyzes the last step of glycolysis, thus decreasing lactic acid production [124]. Accordingly, the cocultivation of cHL cell lines treated with RP6530 and IL-4-stimulated M2 macrophages downregulated the expression of the M2-TAM markers CCL17 and CCL22 [123]. Treatment of HL xenografts with RP6530 shifted the macrophage population towards fewer CD206^+^ and CD301^+^ (M2-TAMs) and more CD86^+^ and MHC-II^+^ macrophages (M1-TAMs) [123].

Altogether, these findings confirm the idea that tumor cells themselves secrete molecules able not only to recruit monocytes but also to induce and maintain an immunosuppressive M2 phenotype [122].

### 5.2. T Cell Polarization towards Immunosuppressive Tregs

Another actor in the cHL immunosuppressive TME is the Treg population, characterized by the expression of CD4, CD25^high^, CD127^low^ (IL-7R), forkhead box protein 3 (FoxP3), cytotoxic T lymphocyte associated protein 4 (CTLA-4), CD73, and CD39, and by an immunosuppressive capacity [125,126]. 

HRS cells educate not only monocytes but also CD4^+^ T cells to become immunosuppressive Tregs. HRS cells may directly recruit Tregs [127], or recruit [37] and then educate CD4^+^ T cells to differentiate into Tregs [107,128] (Figure 1).

Tanijiri et al. [128] demonstrated that CD4^+^ T cells after cocultivation with KM-H2 cHL cells expressed the Foxp3 gene and produced the immunosuppressive cytokine IL-10. Therefore, they postulated that human peripheral CD4^+^ naive T cells are recruited and then converted into CD25^+^ Foxp3^+^ Tregs.

To characterize cHL-infiltrating T cells, Wein et al. [107] compared the global gene expression profile of CD4^+^T cells recovered from cHL lymph nodes with the profiles of corresponding T cells from reactive tonsils. This study revealed that T helper cells from cHL lymph nodes polarized towards a Treg phenotype and HRS cells could induce Treg differentiation [107]. The molecules that may affect the polarization of T helper cells towards Tregs (IL-4, IL-6, IL-15, and PG-E2) are expressed by HRS cells, but also by the TME [17]. To demonstrate that HRS cells can polarize T helper cells, the authors cocultured HRS cells with CD25-depleted CD4^+^ T cells from healthy donors. They found that HRS cells educated T helper cells to become Tregs (CD4^+^ CD25^high^ CD127^low^ FOXP3^+^, CTL4^+^). Coculture with non-Hodgkin lymphoma cells did not increase Treg features, suggesting this is a unique strategy of cHL (Figure 1). 

### 5.3. Education of MSCs

MSCs are bone marrow- or adipose-derived cells that have fibroblast-like morphology after isolation. They are multipotent progenitors capable of differentiating into various cell types, including chondrocytes, osteoblasts, adipocytes, and fibroblasts [129]. Increasing evidence suggests that MSCs contribute to cancer progression [130,131], including cHL [31,110,132]. HRS cells can educate MSCs. Indeed, treatment of MSCs with cHL conditioned medium increased MSC growth, CCL5 secretion, and resistance to the cytotoxic effects of doxorubicin [40]. In turn, educated MSCs augmented HRS cell growth and further attracted monocytes, thus contributing to the formation of an immunosuppressive TME. These results support the idea of MSC involvement as active player in the TME (Figure 1).

### 5.4. Education by Extracellular Vesicles

The education of normal cells without cell–cell contacts implies communication through soluble factors like extracellular vesicles from different cell types, including tumor cells [133]. Extracellular vesicles contribute to the communication with distant sites and modify the function of receiver cells, thus affecting tumor development and progression, immune suppression, angiogenesis, and metastasis formation [133]. 

Hansen et al. [134] demonstrated that HRS cells release membrane-anchored CD30 into the TME. CD30-containing extracellular vesicles, guided by a network of actin- and tubule-based protrusions, stimulated IL-8 release from immune cells. IL-8, by promoting the trafficking of neutrophils and myeloid-derived suppressor cells, promoted immunosuppression [135].

CAFs express and secrete many different tumor components. CAFs produce the extracellular matrix and secrete molecules involved in tumor growth, TME formation, resistance to chemotherapy, and immunosuppression [136]. HRS cells can educate fibroblasts to become CAFs. Dorsam et al. [137] found that HL extracellular vesicles can change the secretome of fibroblasts toward a CAF phenotype. These vesicles were internalized by fibroblasts, which increased their migratory capacity, showed an inflammatory phenotype, and increased expression of alpha-smooth muscle actin, a marker of CAFs. Extracellular vesicle-treated fibroblasts enhanced the release of pro-inflammatory cytokines (e.g., IL-1α, IL-6, and TNF-α), growth factors (G-CSF and GM-CSF), and the pro-angiogenic factor VEGF [137] (Figure 1). These findings were confirmed using a cHL xenograft model [137].

Overexpression of ADAM10, together with increased release of NKG2D ligand (NKG2D-L) and reduced activation of Teffs with anti-tumor cell capacity, has been described in HL [31,110]. Tosetti et al. [138] demonstrated that the mature bioactive form of ADAM10 is released in exosome-like vesicles (ExoV) by HRS cells and lymph node mesenchymal stromal cells (HL MSC). ExoV (ADAM10^+^) released by HRS cells enhanced MIC-A shedding by HL MSCs, while ExoV from HL MSCs induced both TNF-α and CD30 shedding by HRS cells (Figure 1). Thus, the cross-talk between HL MSCs and HRS cells, mediated by ExoV (ADAM10^+^), may result in the release of cytokines (TNF-α) and soluble molecules (sMICA or sCD30) that potentially interfere with host immune responses or with antibody drug conjugate-based immunotherapy like anti-CD30 brentuximab vedotin or iratumumab. Moreover, pretreatment of HL MSCs or HRS cells with the ADAM10 inhibitors LT4 and CAM29 counteracted the ADAM10 sheddase activity carried by ExoV and maintained the cytotoxic effects of brentuximab-vedotin, the anti-CD30 antibody-drug (mauristatin)-conjugate, and the anti-CD30 iratumumab on HRS cells [138]. Thus, ADAM10 inhibitors may counteract the release of molecules that contribute to the immunosuppressive TME [138,139]. 

In conclusion, understanding how HRS cells educate their tumor milieu to sustain tumor growth and exhibit immunosuppressive activity is clinically relevant, and highlights new therapeutic approaches targeting the TME. 

## 6. Targeting the TME to Counteract Its Tumor-Protective Effects 

In cHL, disrupting TME interactions is a goal for immunotherapy and has led to the idea of targeting the tumor and the host as well [6,17,21,27]. As a consequence, new therapeutic strategies have been developed or proposed to not only kill tumor cells, but also to increase the host antitumor immune responses [94,95,96,140], inhibit TME formation, counteract the immunosuppressive programming of both T cells and monocytes, and directly target monocytes [40,123,141]. 

### 6.1. Checkpoint Inhibitors and Adjuvants: Nivolumab, Pembrolizumab, and Indoximod

Despite a great inflammatory infiltrate, patients with cHL have an impaired cellular immune response [107,142]. This is mediated by several factors including the high expression of PD-L1 and PD-L2 ligands by HRS cells, since PD-1 engagement by PD-L1 leads to T cell exhaustion, that is reduced T cell activation and proliferation [2,6] (Figure 1). PD-L1/L2 over-expression by HRS cells is due to gene amplification at the 9p24.1 locus and/or latent Epstein–Barr virus infection [2]. PD-L1 expression in cHL tissues is relatively high, because PD-L1 is also expressed by TAMs, providing a possible explanation for the poor prognosis of patients with a high number of TAMs [100].

**Figure 2:**
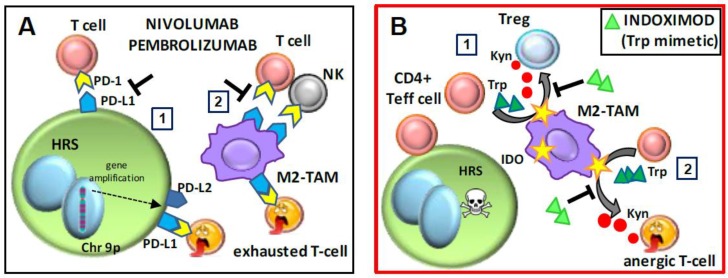
Mechanism of action of the checkpoint inhibitors nivolumab and pembrolizumab, and the tryptophan mimetic indoximod. (**A**) Anti-programmed death-1 (PD-1) antibodies. The anti-PD-1 antibodies (nivolumab and pembrolizumab) (1) inhibit the engagement of PD-1^+^ T cells by PD-L1^+^ Hodgkin and Reed–Sternberg (HRS) cells or PD-L1^+^ M2-tumor-associated macrophages (TAMs), (2) thus counteracting T and natural killer (NK) cell exhaustion due to PD-1/PD-L1 interactions. (**B**) Indoximod. (1) Indoleamine 2,3-dioxygenase (IDO) converts tryptophan (Trp) to kynurenine (Kyn). In classic Hodgkin lymphoma (cHL), indoximod, acting as a Trp mimetic, may inhibit the polarization of effector CD4^+^ T cells towards Tregs due to increased levels of kynurenine. (2) Indoximod may counteract anergy or exhaustion of effector CD4^+^ T cells due to kynurenine. Chr 9p, chromosome 9 petit; Teff, effector T cell.

Nivolumab and pembrolizumab are human IgG4 (S228P) monoclonal antibodies that target PD-1, which is expressed on activated T cells, B cells, and myeloid cells. Both nivolumab and pembrolizumab bind and block engagement of PD-1, thereby activating T cells and cell-mediated immune responses [143] (Figure 2A).

A recent study suggested that the clinical responses to pembrolizumab (anti-PD-1 therapy) might be, at least in part, related to the disruption of TAM–NK cell interactions [101]. The study found that pretreated cHL patients have high CD56-bright, CD16-dim NK cells with high PD-1 expression that returned to normal or low levels after chemotherapy. In vitro experiments demonstrated that an anti-PD-1 antibody counteracted the suppressive activity of PD-L1^+^ macrophages on PD-1^+^ NK cells [101], suggesting that in vivo TAMs may interact via PD-L1 not only with cytotoxic T cells but also with NK cells. Even if in the vast majority of cases a low number of PD-1+ CD4+ T cells was found in cHL tissues [144], a phase 1 trial of nivolumab showed high and durable responses in 23 heavily pretreated patients with relapsed/refractory disease, indicating that immune checkpoint blockade is an effective treatment approach in cHL [2]. A phase 2 trial showed encouraging results leading to approval by the US Food and Drug Administration (FDA) of nivolumab for cHL patients for whom autologous stem cell transplantation and brentuximab vedotin had failed [1,92,145]. 

Pembrolizumab, which has similar activity and effects to nivolumab, was approved by the FDA for cHL relapsed or refractory disease [94,95]. The KEYNOTE-087 trial showed additional data with regard to PD-L1 expression levels in both HRS cells and TAMs [95]. Clinical responses to PD-1 inhibitors were also seen in patients with low levels of the ligand, suggesting that, at least in cHL, only PD-L1 expression is not a good predictive biomarker [95].

Besides the PD1/PDL1 axis, several other molecules are critical regulators of the immune response and may be the target of therapeutic intervention. Lymphocyte activation gene-3 (LAG-3) is an immune checkpoint, largely expressed in the tumor microenvironment of cHL [146]. By interacting with its ligand, MHC class II, LAG-3 plays a negative regulatory role and suppresses T cell function; in combination with PD-1 it mediates T cell exhaustion [147]. These findings provided a strong rationale for their blockade alone or in combination in relapsed/refractory patients with cHL. An ongoing phase 1/2 clinical trial is the testing of the safety, tolerability, and maximum tolerated dose of BMS-986016, an anti-LAG-3 monoclonal antibody, administered alone or in combination with nivolumab to subjects with relapsed or refractory Hodgkin lymphoma (ClinicalTrials.gov Identifier: NCT02061761). 

Another checkpoint molecule that was therapeutically evaluated in cHL, even though only a few clinical trials have been performed, is CTLA4, which competes with CD28 for the binding of CD80 and CD86, thereby antagonizing T-cell activation [2]. Colony-stimulating factor 1 receptor (CSFR-1), the receptor of both M-CSF and IL-34 [148], is expressed on monocytes and on HRS cells and is associated with an increased number of infiltrated TAMs [149]. Two CSFR-1 inhibitors, JNJ-40346527 [150] and PLX3397 [151], exerted low activity in relapsed or refractory cHL. The bispecific antibody against CD30 and CD16A (AFM13) on NK cells did not obtain encouraging results [152]. 

Small-molecule inhibitors of IDO, e.g., epacadostat and navoximod, and the Trp mimetic indoximod are emerging as an additional option to counteract immunosuppression (T cell exhaustion) [153] as immunometabolic adjuvants. IDO inhibitors have been proposed as new agents to be combined with chemotherapy and radiotherapy as standard care in oncology [153,154]. Indoximod is currently being tested in clinical trials in other cancers for its ability to enhance the immune responses triggered by chemotherapy, vaccines or checkpoint inhibitors such as nivolumab, used in refractory or relapsed cHL [153,155]. In cHL, high levels of IDO, expressed by TAMs infiltrating cHL lymph nodes and by vascular endothelial cells [117], positively correlated with the serum Kyn/Trp ratio [116]. The overall survival was significantly shorter for cHL patients with a high Kyn/Trp ratio, suggesting that the evaluation of serum levels of Kyn and Trp may be useful for predicting prognosis [116] and that IDO blockage could have antitumor effects (Figure 2B). 

### 6.2. The CCR5 Antagonist Maraviroc

Maraviroc is a CCR5 antagonist approved by the FDA for the treatment of HIV [156,157]. It was recently repurposed for cancer treatment [158] since it blocks metastasis of basal breast cancer cells [159], reduces metastatic breast cancer growth in the lungs [160], and inhibits the accumulation of fibroblasts in human colorectal cancer (CRC) [161]. In functional organoids derived from metastatic CRC patients, maraviroc polarized macrophages towards an M1-like functional state with antitumor activity [162]. In a phase I trial in patients with liver metastases from advanced refractory CRC, treatment with maraviroc was associated with attenuation of tumor-promoting inflammation within the tumor tissue and with objective tumor responses [162]. 

**Figure 3:**
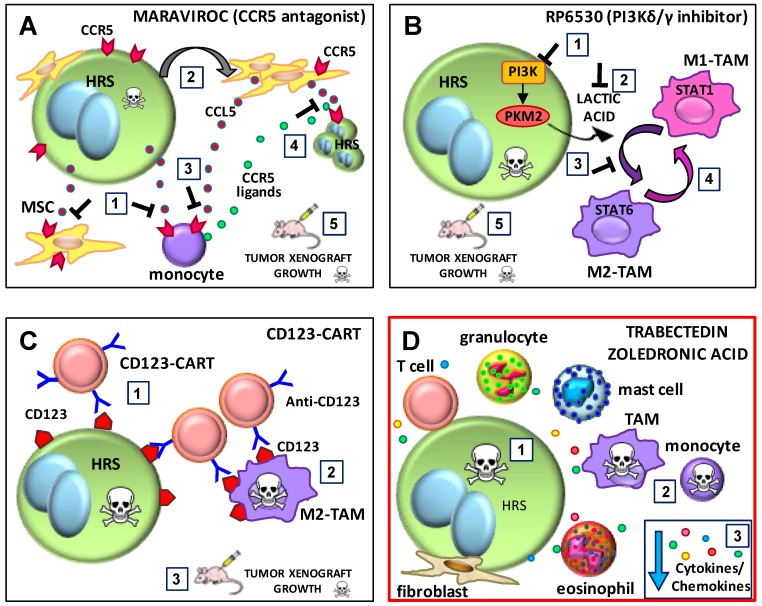
Proposed mechanism for the inhibitory effects of maraviroc, RP6530, chimeric antigen receptor T cells (CART)123, trabectedin and zoledronic acid on tumor cells and the tumor microenvironment (TME). (**A**) Maraviroc. (1) The C-C chemokine receptor type 5 (CCR5) antagonist maraviroc inhibits the recruitment of both monocytes and mesenchymal stromal cells (MSCs) by classic Hodgkin lymphoma (cHL) cells. (2) The education of MSCs cells (E-MSCs) induces the secretion of C-C motif ligand 5 (CCL5) (red dots). (3) Maraviroc inhibits the recruitment of monocytes by CCL5 secreted by E-MSCs. (4) Maraviroc inhibits cHL clonogenic growth promoted by CCR5 ligands (green dots) secreted by tumor-educated monocytes (E-monocytes) and E-MSCs (CCL5+). (5) Maraviroc decreases cHL tumor xenograft growth and monocyte infiltration. (**B**) RP6530, a PI3K-δ/γ inhibitor. (1) RP6530 inhibits Hodgkin and Reed–Sternberg (HRS) cell growth (2) and lactate acid production and secretion. (3) RP630 treatment of HRS cells inhibits their ability to maintain tumor-associated macrophage (TAM)-M2 immunosuppressive polarization. (4) RP6530 repolarizes M2-TAMs towards M1-TAMs. (5) RP6530 decreases cHL tumor xenograft growth and M2-TAM reprogramming. (**C**) CART123. (1) Anti-CD123-CART cells kill CD123^+^ HRS cells and (2) CD123^+^ M2-TAMs. (3) Anti-CD123-CART cells exert potent effector function against Hodgkin lymphoma in vivo. (**D**) Trabectedin and zoledronic acid. (1) Trabectedin and zoledronic acid may kill HRS cells, (2) monocytes and TAMs. (3) Trabectedin and zoledronic acid may decrease the secretion of inflammatory and angiogenic factors by tumor cells or the TME. PI3K, phosphoinositide 3-kinase; PKM2, pyruvate kinase isozymes M2.

Both CCR5 and its ligand CCL5 are constitutively expressed by cHL-derived cell lines [37,39], by tumor cells from cHL lymph nodes, and by bystander cells including stromal cells and lymphocytes [37,77]. The CCR5 receptor expressed by HRS cells is fully functional and CCR5 ligands can work as paracrine [40] and autocrine [37] growth factors. High levels of CCL5 in cHL tumor tissues correlated with poor prognosis and monocyte infiltration [40]. Maraviroc decreased both MSC and monocyte recruitment by HRS cells (Figure 3A) and monocyte recruitment by tumor-educated MSCs (Figure 3A), and it slightly decreased tumor cell growth alone but enhanced doxorubicin and brentuximab vedotin cytotoxic activities [40]. In a heterospheroid model of TME interactions, generated by the three-dimensional cocultivation of HRS cells with MSCs and monocytes, maraviroc counteracted heterospheroid formation and cell viability [40]. In mice bearing cHL tumor xenografts, maraviroc reduced tumor growth by more than 50% and inhibited monocyte accumulation without weight loss (Figure 3A). Therefore, the repurposed drug maraviroc may be a new therapy with fast clinical application in cHL. 

### 6.3. The PI3K-δ/ϒ Inhibitor RP6530

Idelalisib is the first PI3K-δ inhibitor to be approved for follicular lymphoma [163] and chronic lymphocytic leukemia [164]. Recently, it has been demonstrated that the selective targeting of the ϒ isoform of PI3K in TAMs modulates the immunosuppressive TME, resulting in tumor regression [165]. Hyperactivation of the PI3K/AKT pathway is involved in the pathogenesis of cHL [8]. Idelalisib, which can kill HRS cells [166], was approved for a phase II study of relapsed or refractory cHL [167]. Because the δ and ϒ isoforms of PI3K are overexpressed in both HRS cells and the TME, Locatelli et al. [123] proposed that the PI3K-δ/ϒ inhibitor RP6530 might affect both HRS cells and the TME. They demonstrated that RP6530 inhibits the growth of HRS cell lines and, by decreasing lactate production by tumor cells, it shifts the tumor activation of macrophages from an immunosuppressive M2-like phenotype to an inflammatory M1-like condition (Figure 3B). Treatment of M2-polarized macrophages with RP6530 re-shaped them to an inflammatory M1-like state (Figure 3B). These in vitro studies were confirmed in vivo. Indeed, in cHL tumor xenografts, RP6530 repolarized TAMs into proinflammatory macrophages and inhibited tumor vasculature formation, leading to tumor regression. In a phase I trial with RP6530, patients with HL showed good responses (partial or complete) associated with a significant inhibition of circulating myeloid-derived suppressor cells and a significant reduction of CCL17 levels [123].

### 6.4. CD123-CAR T Cells

A new immunotherapeutic approach for the treatment of malignant hematological diseases is reprogramming autologous T cells with chimeric antigen receptor T cells (CAR T cells) [168]. Briefly, autologous T cells are genetically altered by the addition of a chimeric antigen receptor (CAR) that specifically recognizes cancer cells. The resulting CAR T cells are then re-infused into the patient to attack the tumor [168]. CAR T cells received FDA approval for the treatment of relapsed juvenile B-ALL and DLBCL and are currently being evaluated in additional diseases including cHL [168]. 

The CD30 antigen is considered the most promising target antigen for CAR T cell approaches in cHL, and preliminary in vitro and in vivo experiments have revealed encouraging results [168]. Unfortunately, CAR T cells can be destroyed by M2-TAMs [60]. CD123, α chain of the IL-3 receptor, is a dendritic marker expressed on HRS cells, in up to 60% of cases, and on TAMs [48,60,169]. Considering that CAR T cells are destroyed by M2-TAMs, and that M2-TAMs express CD123, Ruella et al. [60] developed CD123-CAR T cells (Figure 3C). These cells targeted not only HRS cells, but also CD123-expressing M2-TAMs. Experiments with immunodeficient mouse models demonstrated that CART123 eliminate Hodgkin lymphoma and established long-term immune memory [60] (Figure 3C).

### 6.5. Trabectedin and Zoledronic Acid

Trabectedin and zoledronic acid are two drugs able to kill both tumor cells and TAMs, and might therefore be new cHL therapies (Figure 3D). 

Trabectedin (ET-743, Yondelis) is a marine alkaloid isolated from the tunicate *Ecteinascidia* [141]. Approved in Europe as second-line therapy for soft tissue sarcoma, ovarian cancer, leiomyosarcoma and liposarcoma [141], trabectedin is under evaluation in hematological malignancies [170,171,172]. Trabectedin affects tumor cells as well as the TME, given that selective monocyte and TAM targeting and reduction are key components of its anticancer activity [141] (Figure 3D). It binds DNA covalently and blocks active transcription, reducing the secretion of pro-inflammatory and pro-angiogenic molecules; it interferes with DNA repair efficiency, leading to DNA double strand breaks and cell cycle blockade [141].

Zoledronic acid is a biphosphonate used to treat osteoporosis and to reduce pain from bone metastases during adjuvant therapy for solid cancers [173]. Zoledronic acid is a potential therapy to reduce cancer growth and the supportive role of the TME. Both zoledronic acid and its liposomal form significantly affect the secretion of CCL5 and IL-6 in MSCs [174,175], suggesting that it could exhibit antitumor activity by affecting the ability of MSCs to interact with tumor cells and to recruit monocytes to the TME [40,174]. In cellular models of prostate cancer, zoledronic acid decreased M2 macrophage polarization, inhibited the activation of normal fibroblasts by M2 macrophages, and reverted the activation of CAFs [176]. 

## 7. Conclusions

New molecular techniques have allowed investigations into the characteristic genetic lesions, pathway dependencies, and immune escape mechanisms in cHL. However many questions remain about the mechanisms involved in cHL TME building, the education of normal cells in the TME, and the roles of different cell types and molecules during the course of the disease. The main challenge is to translate and apply all the information in the clinic and provide the rationale to find new prognostic factors and better risk stratification schemes, to choose less toxic treatments or repurposed drugs to not only target cancer cells but also disrupt TME interactions and reprogramming of immunosuppressive cells.

## Figures and Tables

**Table 1:** Cytokine and chemokine expression, induction and function in Hodgkin lymphoma.

Cytokine	Expression by HRS (Tissue or Cell Lines)	Expression (TME) and Induction in Normal Cells	Function in cHL
**IL-3**	Absent	Expressed by T cells and eosinophils [45] Induced in T cells by HRS cells [46]	Upregulation of CD40L and CD30L in eosinophils [47]; proliferation of eosinophils and mast cells [46]; proliferation of HRS cells [48]
**IL-5**	Tissue [49] Cell lines [50]	Expressed by T cells [45]	Eosinophil recruitment [23]; upregulation of CD40L and CD30L in eosinophils [47]
**IL-7**	Tissue [51] Cell lines [52]	Expressed by T cells [45] and HL fibroblasts [52]	Growth factor for Treg cells; proliferation of HRS cells; IL-7 stimulates IL-6 secretion by HL fibroblasts [52]
**IL-13**	Tissue [53] Cell lines [53]	Rarely expressed by small lymphocytes [53]	Growth factor for fibroblasts [30]; autocrine growth factor for HRS cells [53]
**IL-15**	Tissue [54] Cell lines [54,55]	Expressed by monocytes, dendritic cells, endothelial cells [54]	Proliferation, survival, and apoptosis resistance of HRS cells [54]
**APRIL**	Tissue [56] HL cell lines [56]	Neutrophils [56,57]	Proliferation of HRS cells [56,57]
**FGF-2**	Tissue [58] Cell lines [59]	Expressed by stromal cells and histiocytes [58]	Growth factor for fibroblasts [30] and MSCs [40], endothelial cell tubulogenesis [32,59]
**GM-CSF**	Tissue [60] Cell lines [60,61]	Likely in activated T cells, B cells, macrophages, mast cells, endothelial cells and fibroblasts [62]	Recruitment of eosinophils [23]; up-regulation of CD40L and CD30L in eosinophils [47]; M2-TAM differentiation [60]
**Jagged-1**	Tissue [63] Cell lines [59]	Endothelial cells, smooth muscle cells and epithelioid cells [63]	Proliferation and survival of HRS [63]
**LT-α**	Tissue [64] Cell lines [65]	Extracellular stroma [66]	Activates endothelial cells to enhance T cell recruitment [66]
**M-CSF**	Tissue [67] Cell lines [40,60,68]	Endothelial cells and fibroblasts [67]	Recruitment and proliferation of monocytes; differentiation of M2-TAM [40,69]
**TGF-β**	Tissue [70] Cell lines [59]	T lymphocytes; [71] eosinophils [72] Induced in monocytes by HRS [40]	Growth factor for fibroblasts [30] and MSCs [40]; endothelial cell tubulogenesis [32,59]
**TNF-α**	Tissue [64] Cell lines [59]	Lymphocytes and macrophages [73]	Growth factor for fibroblasts [30] and MSCs [40]; induction of eotaxin secretion by fibroblasts [74]
**VEGF**	Tissue [75] Cell lines [32,59]	Macrophages and lymphocytes [75,76]	Endothelial cell tubulogenesis [32,59]
**CCL3 (MIP-1α)** **CCL4 (MIP-1β)**	Low levels or absent	Macrophages [77] Increased in monocytes by HRS [40]	Proliferation of HRS [40]
**CCL5 (RANTES)**	Tissue [40,78] Cell lines [37,39,78]	T cells and B cells [77] Induced in MSCs [40] and fibroblasts [37] by HRS cells, or increased by cultivation of HRS cells with fibroblasts [79]	Recruitment of monocytes/macrophages and MSCs [40], eosinophils and T cells [37], and mast cells [39]; proliferation of HRS cells [37,40]
**CCL11 (Eotaxin)**	absent	Fibroblasts and some macrophages [45,74] and smooth muscle cells [80] Induced in fibroblasts by TNF-α secreted by HRS cells [74]	Recruitment of eosinophils and T cells by tumor fibroblasts [74]
**CCL17 (TARC)**	Tissue [42] Cell lines [42,59,81]	Occasional in macrophages [82] Induced in monocytes by HRS cells [40]	Recruitment of T cells and Tregs [42]
**CCL20 (MIP-3α)**	Tissue [41] Cell lines [41]	Some neutrophils [41]	Recruitment of Tregs [41]
**CCL22** **(MDC)**	Tissue [43,83] Cell lines [78,80,84]	Rare in histiocytes and endothelial cells (weak cytoplasmic staining) [83]	Recruitment of Th2 and Tregs [43]
**CCL28** **(MEC)**	Tissue [38] Cell lines [38]	Occasionally in TME [38]	Recruitment of eosinophils [38]

APRIL, a proliferation-inducing ligand; FGF, fibroblast growth factors; GM-CSF, granulocyte macrophage colony-stimulating factor; HL, Hodgkin lymphoma; HRS, Hodgkin Reed-Sternberg; IL, interleukin; LT-α, lymphotoxin alpha; MDC, macrophage-derived chemokine; MEC, mucosae-associated epithelial chemokine; MIP, macrophage inflammatory protein; MSC, mesenchymal stromal cells; RANTES, regulated upon activation, normal T cell expressed and secreted; Treg, regulatory T cell; TAMs, tumor-associated macrophages; TARC, thymus and activation regulated chemokine; Th2, T helper cells; TNF, tumor necrosis factor; VEGF, vascular endothelial growth factor.
